# The Effect of Transcutaneous Electrical Acupoint Stimulation on Postoperative Catheter-Related Bladder Discomfort in Patients Undergoing Transurethral Resection of the Prostate

**DOI:** 10.1155/2021/6691459

**Published:** 2021-02-04

**Authors:** Dongdong Liang, ShenHui Jin, LeDan Huang, YeLong Ren, ZhongHeng Du, Li Wang, Ying Ren, KeNing Yang, JunLu Wang, JinGui Yu

**Affiliations:** ^1^Department of Anesthesiology, Qilu Hospital, Cheeloo College of Medicine, Shandong University, Jinan, Shandong 250012, China; ^2^Department of Anesthesiology, First Affiliated Hospital of Wenzhou Medical University, Wenzhou, Zhejiang 325000, China

## Abstract

**Background:**

Catheter-related bladder discomfort (CRBD), an extremely distressing complication secondary to an indwelling urinary catheterization, is frequently reported in patients with transurethral resection of the prostate (TURP), postoperatively. A prospective, randomized, controlled, double-blind study was designed to assess the efficacy of transcutaneous electrical acupoint stimulation (TEAS) as a treatment for CRBD in patients undergoing TURP.

**Methods:**

Seventy benign prostatic hyperplasia male patients undergoing TURP under general anesthesia requiring intraoperative urinary catheterization were enrolled for the trial. An experienced acupuncturist performed TEAS for 30 minutes before general anesthesia with acupoints RN7, RN6, RN5, RN4, and RN3 and bilateral BL32, BL33, and BL34. Mean arterial pressure (MAP), heart rate (HR), oxygen saturation (SPO2), body temperature (T), and blood samples were collected during the surgery. A series of assessments included the incidence and severity of CRBD, postoperative pain, nausea and vomiting, and physical and mental state measurements.

**Results:**

The incidence of CRBD was significantly lower in TEAS group than in control group at the time T5 [9(26%) vs. 28(80%), *P* < 0.001], T9 [20(57%) vs. 28(80%), *P*=0.039], T11 [7(20%) vs. 31(89%), *P* < 0.001], and T12 [4(11%) vs. 7(20%), *P*=0.003]. The severity of CRBD was significantly lower in TEAS group than in control group at the time T5 [0 vs. 10 (29%), *P* < 0.001], T9 [2(6%) vs. 10(29%), *P*=0.011], and T11 [0 vs .9(26%), *P*=0.002]. The QoR-40 total score was higher in TEAS group at time T11 [191.7(4.4) vs. 189.1(4.3), *P*=0.007] and T12 [195.3(1.9) vs. 193.3(3.0), *P* < 0.001]. The postoperative analgesia requirement was higher in control group [5.0(2.9) vs. 3.8(1.9), *P*=0.045].

**Conclusions:**

TEAS could significantly prevent the incidence and severity of CRBD, reduce the postoperative analgesic requirement in the early postoperative period, and promote the quality of early recovery in patients undergoing TURP.

## 1. Introduction

Urinary catheterization is an extensively used intervention during transurethral resection of the prostate (TURP) frequently leading to a series of discomfort postoperatively. Catheter-related bladder discomfort (CRBD), a distressing complication secondary to an indwelling urinary catheterization, is clinically characterized by a burning sensation at the urethra with an urge to void, urinary urgency, the urinary frequency with or without urge incontinence, or painful discomfort in the suprapubic region [1]. The clinical presentation of CRBD mimics those of an overactive bladder (OAB) [2]. Because CRBD is hugely distressing to the patient, it is usually associated with behavioral responses such as postoperative agitation and aggravated pain, delirium, restlessness, and attempting to pull out the urinary catheter, thus, decreasing the quality of postoperative recovery [3, 4]. As the severity of CRBD was recorded to be moderate or severe in 44–60% of patients with indwelling bladder catheters, it necessitates clinical intervention. The involuntary contraction of the bladder mediated by M2 and M3 muscarinic receptors [5] represents the predominant cause of CRBD. Recently, some drugs including tramadol [6], ketamine [7], gabapentin [8], pregabalin [9], and muscarinic receptor antagonist tolterodine [10] have been shown to reduce the incidence and severity of postoperative CRBD. However, the adverse events such as postoperative nausea, vomiting, dry mouth, sedation, dysuria, and constipation have prevented their routine use in the management of CRBD. Thus, to improve and expedite patients' recovery following TURP, an effective and safe treatment strategy is highly desirable.

Transcutaneous electrical acupoint stimulation (TEAS) as a noninvasive and nonpharmacological adjunctive intervention has been extensively used in perioperative anesthesia and postoperative analgesia, owing to its advantages of alleviating preoperative anxiety, relieving postoperative pain, and improving postoperative analgesic effect [11, 12]. Recently, Wang et al. [13, 14] have also shown that TEAS could reduce opioid consumption and alleviate postoperative adverse event such as pain, nausea, and vomiting in patients. Accumulating studies have also indicated that TEAS could reduce the blood lactic acid and serum creatine kinase levels after quantitative load, thus, promoting subsequent recovery of muscle function, improving the state of athletes' body function, and exhibiting a significant antifatigue effect [15, 16].

However, TEAS could prevent the incidence and severity of postoperative CRBD and expedite patients' postoperative recovery in patients undergoing TURP. Therefore, we conducted a randomized controlled clinical trial (RCT) to evaluate the effects of TEAS on the severity of CRBD and changes in the blood lactic acid and blood glucose levels and postoperative recovery in patients undergoing TURP.

## 2. Methods and Materials

This prospective, randomized, controlled, double-blind study was approved by the ethics committee for clinical research in Clinical Research of the First Affiliated Hospital of Wenzhou Medical (no. 2019-111) and registered in the Chinese Clinical Trial Registry (Registration number:ChiCTR1800019951), on December 9, 2018. This trial was performed in accordance with the Declaration of Helsinki. Written informed consent was obtained from each participant.

### 2.1. Patient Population

Eighty male patients undergoing TURP under general anesthesia requiring intraoperative urinary catheterization were enrolled for the trial between December 2019 and August 2020. Inclusion criteria were as follows: patients aged 18–64  years, American Society of Anesthesiologists (ASA) status II or III, clinical diagnosis of benign prostatic hyperplasia, and patients with autonomous writing ability. Exclusion criteria were as follows: severe comorbidities including preexisting severe cardiopulmonary disease, bleeding disorder, hepatic dysfunction, renal impairment, giant/multiple bladder stones, peptic ulcer, and diabetes mellitus, a history of open prostatectomy, unwillingness to participate in the study, a history of urethral stricture, urodynamic examination combined with neurogenic bladder inflammation, prostate tumors, a history of rectal biopsy to determine prostate tumors or pathological findings, severe cognitive impairment, and previous history of acupuncture in the urinary tract.

### 2.2. Randomization

Eligible patients were randomly distributed into two groups, with the help of a computer-generated table of random numbers by an independent statistician. An anesthesiologist (RYL) performed the preoperative evaluation and recorded the data as participants had signed the consent. Clinical trial consent and QoR-40 (quality of recovery 40 items) questionnaire were explained to the patients one day before the surgery. The independent statistician created identical sealed envelopes before surgery containing either TEAS stimulus (T group) or control group (C group). The acupuncturist (DZH) opened the envelope and performed TEAS. One preoperative nurse (RY), who was not involved in patient care, prepared the items of arterial puncture and TEAS device. An anesthesiologist (HLD) performed the arterial puncture. An anesthesiologist (LDD), who was not aware of the allocation, performed general anesthesia and all intraoperative data recording, and another investigator (WL), in charge of all postoperative assessments, was also blinded to the group identity. Two investigators (YKN and JSH) performed data recording and analysis.

### 2.3. TEAS Protocol

**Figure 1:**
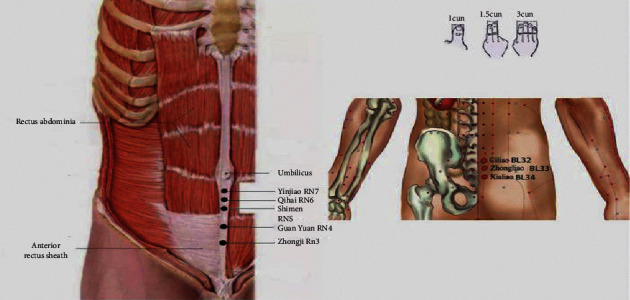
Chinese traditional acupoints location. 1 cun represents the width of the first joint of the thumb in patient. 1.5 cun represents the width of the second stripe of the index finger and middle finger in patients. 3 cun represents the sum of the width of the second joint of the index finger, middle finger and ring finger, and the first joint of the small finger in patient. Yinjiao (RN7), 1 cun below the umbilicus; Qihai (RN6),1.5 cun below the umbilicus; Shimen (RN5), 2 cun below the umbilicus; GuanYuan (RN4), 3 cun below the umbilicus; Zhongji point (RN3), 4 cun below the umbilicus; bilateral ciliao (BL32), second posterior sacral hole, away from the midline by 2 cm, 1.3 cm–1.5 cm below the posterior superior iliac spine; zhongliao (BL33), third sacral posterior hole, 2 cm below the ciliao point, away from the midline about 2 cm; xialiao (BL34), fourth sacral posterior hole, 1.5 cm below the ciliao point, away from the midline about 1 cm.

According to the theory of traditional Chinese medicine [17], we selected Yinjiao (1 cun below the umbilicus, RN7), Qihai (1.5 cun below the umbilicus, RN6), Shimen (2 cun below the umbilicus, RN5), GuanYuan (3 cun below the umbilicus, RN4), Zhongji point (4 cun below the umbilicus RN3), bilateral ciliao (second posterior sacral hole, BL32), zhongliao (a third sacral posterior hole, BL33), and xialiao (fourth sacral posterior hole, BL34) as the acupuncture points (Figure 1). TEAS was applied to the patients through self-adhesive cutaneous electrode pads (5  cm × 5  cm). We used HANS-200A Acupoint Stimulator for TEAS. The frequency was set 2/100 HZ for 30 minutes, the highest tolerable level that caused no discomfort to the patient; in control group, the patients received no stimulation.

### 2.4. Anesthesia and Perioperative Procedures

Considering the comfort of general anesthesia and the patient's active choice of general anesthesia due to fear of surgery, we chose laryngeal mask general anesthesia. All patients were induced with propofol 2 mg／kg and sufentanil 0.3–0.5 *μ*g／kg and intubation was performed with Cisatracurium 2 mg／kg. Anesthesia was maintained by a target-controlled infusion (TCI) of propofol and remifentanil. The bispectrum index (BIS) was maintained within the range of 45–55 by adjusting to control the depth of anesthesia. Mechanical ventilation was performed to maintain PetCO_2_ at 35–40 mmHg. Sufentanil 0.1 mg／kg per 30 minutes during the surgery was administered to provide analgesia. Intravenous prophylactic ondansetron (8 mg) was administered to prevent postoperative nausea and vomiting. After spontaneous breath recovery and consciousness, the neuromuscular blockade was reversed with neostigmine, the trachea was extubated, and patients were moved to the postanesthesia care unit (PACU).

### 2.5. Data Collection

Mean arterial pressure (MAP), heart rate (HR), oxygen saturation (SPO2), and body temperature (T) were recorded before anesthesia (T0), induction (T1), intubation (T2), the onset of surgery (T3), 30 minutes after surgery (T4), at the end of surgery (T5), extubation (T6), out of operation room (T7), at the time of entry to PACU (T8), and at the time out of PACU (T9).

Blood glucose and lactic acid levels were investigated at T0, T4, T5, and T9 and at the time of postoperative 2 hours (T10) by arterial gas analysis. Blood samples were collected and centrifuged at 3500g for 10 minutes and transferred into polyethylene tubes and then stored at −80°C until further analysis. The concentrations of tumor necrosis-*α* (TNF-*α*), interleukin-1 (IL-1), and *β*-endorphin (*β*-EP) were assessed by enzyme-linked immunosorbent assay kit (ELISA kit, Shanghai Westin Biological Technology Co., Ltd.) following manufacturers' protocol.

### 2.6. Primary Evaluation

CRBD was evaluated at the end of surgery (T5), at the time out of PACU (T9), postoperative 24 hours (T11), and postoperative 48 hours (T12). The severity of CRBD was recorded on a 4-point severity scale:1 = none, no discomfort, even on asking; 2 = mild, reported by the patient only on questioning; 3 = moderate, reported by a patient without being questioned; and 4 = severe, reported by the patient without being questioned with behavioral responses.

### 2.7. Second Evaluation

The validated quality of recovery-40 (QoR-40) [18] Chinese version with five domains was used to measure at different time points, including before anesthesia (T0), postoperative 24 hours (T11), and postoperative 48 hours (T12). QoR-40 was comprised of five subscales: physical comfort (PC), emotional state (ES), physical independence (PI), psychological support (PS), and pain (P). Each item was rated on a scale of 1–5, providing a minimum score of 40 and a maximum of 200. A qoR-40 questionnaire was used to measure physical condition after anesthesia in patients.

Patient cognitive function was assessed using the minimum mental state examination (MMSE) (on a scale from 0 to 30, with higher scores indicating better cognition) at time of before anesthesia (T0), at the time out of PACU [T9], postoperative 24 hours (T11), and postoperative 48 hours (T12).

Patients' self-rated intensity of postoperative pain and postoperative nausea and vomiting (PONV) were used to assess verbal rating scales (VRS) and nausea verbal scale description (NVSD) at the end of surgery (T5), at the time out of PACU (T9), postoperative 24 hours (T11), and postoperative 48 hours (T12). VRS was scored as 5 : 0 = no pain, 1 = slight pain, 2 = moderate pain, 3 = severe pain, 4 = very severe pain, and 5 = intolerable pain. NVSD was defined as 0 = no nausea and vomiting, 1 = nausea only, and 2 = vomiting or with severe nausea and vomiting.

### 2.8. Sample Size and Statistical Analysis

The sample size was calculated based on a preliminary experiment (8); approximately 70% of postoperative patients complained of CRBD. We calculated that 23 patients were required in each group to decrease the incidence to 40% after TEAS, assuming a two-sided type I error (*α*) of 5% and a power of 80%. Considering an estimated 20% dropout rate and enabling better statistical power for analyses, 70 patients in total were needed in this study (35 per group).

Statistical analyses were performed using SPSS v 22.0 (SPSS Inc., Chicago, Illinois, USA). Continuous variables were presented as mean and were compared using Student's *t*-test. Dichotomous variables were presented as number *χ*^2^ or Fisher's exact test as appropriate. Categorical variables were presented as proportions of patients and analyzed using the chi-square test. A *P*-value of less than 0.05 was considered statistically significant.

## 3. Results

**Figure 2:**
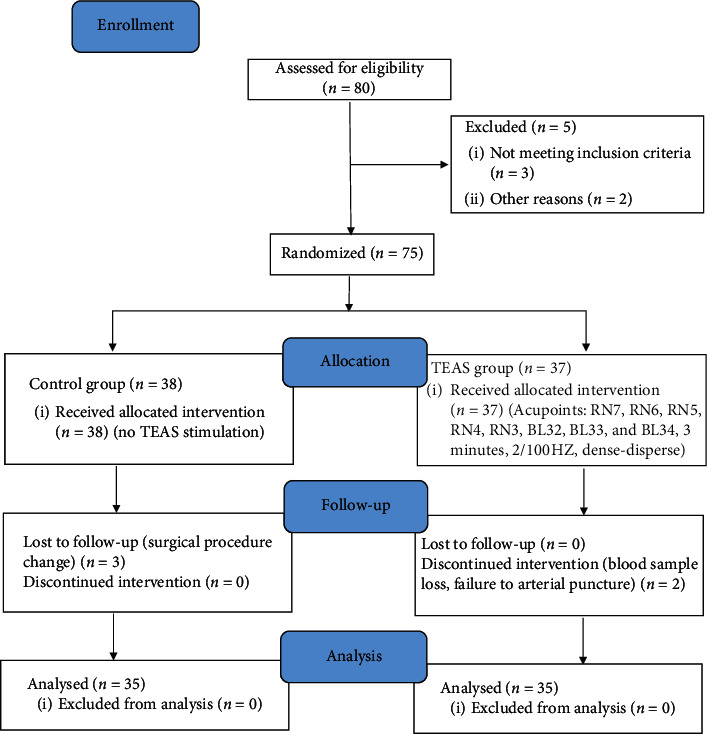
The flow diagram of study procedures. TEAS, transcutaneous electrical acupoint stimulation; Yinjiao (RN7); Qihai (RN6); Shimen (RN5); GuanYuan (RN4); Zhongji point (RN3); bilateral ciliao (BL32); bilateral zhongliao (BL33); and bilateral xialiao (BL34).

Patient characteristics: 80 patients undergoing TURP under general anesthesia were enrolled for this study. Ten patients were excluded due to changes in the surgical procedure, blood sample lost, and loss to follow-up. Finally, 70 male patients were included in the study, and all the data were collected and analyzed (Figure 2). There were no significant differences between the two groups for age, heights, weight, body mass index (BMI), time of anesthesia and surgery, intraoperative sufentanil, propofol, remifentanil consumption, and liquid dosage (Table 1).

### 3.1. Primary Outcomes

Patients in TEAS group had a significantly lower incidence of CRBD than those in control group at time T5 [9(26%) vs. 28(80%), *P* < 0.001], T9 [20(57%) vs. 28(80%), *P*=0.039], T11 [7(20%) vs. 31(89%), *P* < 0.001], and T12 [4(11%) vs. 7(20%), *P*=0.003]. The incidence of moderate to severe CRBD in TEAS was significantly lower than that of the control group at time T5 [0 vs. 10(29%), *P* < 0.001], T9 [2(6%) vs. 10(29%), *P*=0.011], and T11 [0 vs. 9(26%), *P*=0.002] (Table 2).

### 3.2. Secondary Outcome Measure

Intraoperative vital signatures: values of MAP and HR SPO2 and core temperatures changed over time in the TEAS group and control group during the surgery. However, neither MAP and HR nor SPO2 and core temperatures between the two groups had significant differences at all times (Table 3).

### 3.3. Postoperative Pain and PONV and QoR-40 Scores

There was no significant difference in the incidence of PONV and pain in patients over time between the two groups. However, the number of patients who required extra analgesia in the control group was much higher than that in the TEAS group [3.8(1.9) vs. 5.0(2.9), *P*=0.045] (Table 4).

The QoR-40 total scores at the time T11 [191.7(4.4) vs. 189.1(4.3), *P*=0.007] and T12 [195.3(1.9) vs. 193.3(3.0), *P* < 0.001] were significantly higher in the TEAS group than those in the control group. MMSE score decreased at T9 compared with T0 [28.1(1.4) vs. 26.2(2.8)], T11 [28.1(1.6) vs. 26.2(2.8)], and T12 [28.3(1.4) vs. 26.2(2.8)]; however, there was no significant difference between the two groups (Table 4).

### 3.4. Serum Biochemical Parameters Assessment

Serum levels of glucose increased from the time T5 to T10, which corresponded with the usage of 5% glucose solution during the surgery by 57.7% in the TEAS group and 58.3% in the control group; however, no significant difference was observed between the two groups. The serum concentration of IL-1, TNF-*α*, *β*-EP, and lactic acid barely altered at the time from T0 to T10; however, there was no significant difference between the groups (Table 5).

## 4. Discussion

By the theory of traditional Chinese medicine, acupuncture meridians represent “channels” through which energy called “meridian qi” flows. Acupuncture is a technique for balancing the flow of energy. This energy is believed to flow through corresponding acupoints and the meridians or pathways in the human body [19]. RN7, RN6, RN5, RN4, and RN3 belong to Ren channel, which often can treat the lower abdomen, urinary and reproductive system, and other diseases, through different ways to stimulate these acupoints that can play the role of reinforcing the vital essence and strengthening the primordial qi, tonifying the lower coke, nourishing the liver and spleen, promoting blood circulation and stasis, and so on. Bilateral ciliao (BL32), zhongliao (BL33), and xialiao (BL34) belong to the foot-sun bladder meridian, and these meridians and acupoints mainly treat diseases associated with the urinary system by traditional Chinese medicine. In this context, Sun et al. [20] reported that acupuncture on BL32, BL33, and BL34 for one time per day for about 4 days could markedly improve urinary incontinence in a patient undergoing hysterectomy; moreover, the patient recovered to a normal urinary frequency. In yet another clinical trial [21], patients undergoing OAB received real acupuncture with BL32, GuanYuan (RN4), Shenshu (BL23), Sanyinjiao (SP6), Taixi (KI3), Weiyang (BL39), and Pangguang Shu (BL28). The results demonstrated that acupuncture could improve daily micturition and symptoms of OAB, including incontinence and urgency episodes and voided volume per micturition episode. These studies suggested that acupuncture points could produce synergetic effects on urinary function in patients undergoing TURP and contribute to the TEAS-induced beneficial effects in this study; considering these studies, we have chosen these acupoints.

CRBD is distressing complication and is frequently neglected and left untreated due to some different underlying mechanisms involved. The physiological and pathological mechanism in a patient with bladder diseases is the loss of bladder neurological function, which is caused by cholinergic innervation from the pelvic nerves and adrenergic innervation from the hypogastric nerves. Detrusor muscle contraction and activity of inflammatory mediators due to catheterization can promote prostaglandin (PG) synthesis, which may play a critical role in the occurrence of inflammation. It has been testified that electroacupuncture (EA) could inhibit the expression of PG E2 to relieve pain [22]. Possible involvement of anti-inflammation by EA has implicated that EA could activate sympathetic nerve by splanchnic nerve activity transmitted to celiac ganglia at pathological responsiveness in internal organs [23]. On the other side, bladder muscle involuntary contraction triggered by muscarinic receptors especially type M3 was considered to be responsible for symptoms of CRBD [24]. The antimuscarinic blockers reported a beneficial effect to prevent the incidence of CRBD. However, various agents side effects such as dry mouth and sedation get along with the efficacy. A study have shown that EA could considerably downregulate the expression levels of M3 in irritable bowel syndrome rats [25], which showed a beneficial nondrug therapeutic method. Besides, the neural anatomy illustrated that sensation of the bladder triangle and urethra arises from the pudendal nerve derived from sacral nerves S2–S4 [26]. BL32, BL33, and BL34 are on the course of posterior branch of the second, third and fourth sacral nerves, respectively. Electrical stimulation of BL32, BL33, and BL34 can increase the maximum pressure of urethral closure by stimulating the sacral nerve more easily. A clinical trial [27] reported that bilateral pudendal nerve block could significantly decrease the incidence of CRBD and control the degree of CRBD severity at a low level. Our results are in line with those studies suggesting that TEAS possibly stimulated sacral nerves and pelvic muscles to regulate the function of the bladder, urethral sphincter, and nerve innervation effector *via* related acupuncture points [25].

Besides, patients in the TEAS group showed a better recovery at postoperative 24 hours and 48 hours. Previous studies have demonstrated that TEAS twice with bilateral zusanli (ST36) and Sanyinjiao (SP6) could shorten the time of first flatus, the time to first oral liquid and solid intake, and the postoperative hospital stay and reduced postoperative complaints [28, 29]. In addition, another study [30] showed that 14 sessions of moxibustion on participants undergoing depression could significantly adjust depression-related fatigue and increase the safety of the treatment. These findings supported that TEAS might positively regulate the inner immune system and balance physiological functions between yin and yang or between qi and blood in the body according to traditional Chinese medicine. In our study, both physical (QoR-40 scores) and mental status of patients (MMSE scores) rapidly ascended to the preoperative status with pretreatment of TEAS treatment.

Furthermore, measurement of serum biochemical parameters to assess the surgical stress indicated no significant differences between the TEAS group and the control group. The increase in blood glucose values after surgery might be attributed to the use of 5% glucose solution perioperatively. Surprisingly, the levels of IL-1, TNF-*α*, *β*-EP, and blood lactic acid remain unaltered at different observation time between the two groups. These objective data suggested that patients in the two groups did not experience intense surgical stress and TEAS provided a stable internal environment for recovery in patients. More importantly, it was relatively easier to administer TEAS and perform follow-up assessments.

There are certain limitations to this study. First, this study is a clinical surgical study; surgeons cannot be completely unaware of the intervention, but they do not know which patients have received TEAS intervention. However, the anesthesiologist, acupuncturist, and follow-up investigator were completely blinded to the data recording and postoperative assessments. Second, invasive artery puncture may cause vascular damage to patients, but the whole process is operated by a skilled anesthesiologist and stopped after more than two attempts. Despite the limitations, we assessed the status of surgery in patients using questionnaires on MMSE and QoR40, providing a steady condition for TEAS intervention by the serum biochemical parameters. This study provides a safe and comfortable condition for patients.

## 5. Conclusions

In summary, the findings of this study indicated that TEAS could significantly prevent the incidence and severity of CRBD, reduce the postoperative analgesic requirement in the early postoperative period, and promote the quality of early recovery in patients undergoing TURP. Collectively, the study suggested that TEAS has the benefits of being noninvasive, the nonpharmacological modality for perioperative analgesia, and early postoperative recovery and can be implemented in clinical practice.

## Figures and Tables

**Table 1:** Patient characteristics and intraoperative anesthetic dosage.

	TEAS group (*n* = 35)	Control group (*n* = 35)
Age (year)	70.8 (6.5)	69.1 (6.1)
Height (cm)	164.3 (6.2)	165.3 (6.1)
Weight (kg)	63.8 (7.5)	63.2 (8.7)
BMI	23.6 (2.5)	23.1 (2.6)
Surgery time (min)	55.6 (20.4)	52.2 (18.5)
Anesthesia time (min)	69.4 (19.1)	66.6 (16.9)
Intraoperative fluids (ml)	874.3 (243.0)	814.3 (245.1)
Sufentanil consumption (ug)	21.4 (3.1)	20.0 (2.9)
Remifentanil consumption (ug)	521.5 (206.8)	464.7 (156.0)
Propofol consumption (mg)	250.7 (99.9)	225.9 (74.8)
Cisatracurium consumption (mg)	7.2 (1.7)	7.4 (1.7)

Data is presented as mean standard deviations. TEAS: transcutaneous electrical acupoint stimulation.

**Table 2:** The incidence of postoperative catheter-related bladder discomfort between two groups.

Time (hours)	T5		T9		T11		T12	
Group	Control (*n* = 35)	TEAS (*n* = 35)	Control (*n* = 35)	TEAS (*n* = 35)	Control (*n* = 35)	TEAS (*n* = 35)	Control (*n* = 35)	TEAS (*n* = 35)
CRBD	28 (80%)	9 (26%)^*∗*^	28 (80%)	20 (57%)^*∗*^	31 (89%)	7 (20%)^*∗*^	19 (54%)	4 (11%)^*∗*^

*Severity*								
Mild	18 (51%)	9 (26%)	18 (51%)	18 (51%)	22 (63%)	7 (20%)	19 (54%)	4 (11%)
Moderate	10 (29%)	0^*∗*^	10 (29%)	2 (6%)^*∗*^	8 (23%)	0^*∗*^	0	0
Severe	0	0	0	0	1 (3%)	0	0	0

Data is presented as the number (%) of patients. CRBD: catheter-related bladder discomfort; TEAS: transcutaneous electrical acupoint stimulation. T5: at the end of surgery; T9: at the time out of PACU; T11: postoperative 24 hours; T12: postoperative 48 hours. ^*∗*^*p* < 0.05 vs. control group.

**Table 3:** Intraoperative vital signs of patients in two groups.

	TEAS group (*n* = 35)				Control group (*n* = 35)			
	MAP (mmHg)	HR (bpm)	SPO_2_ (%)	T (°C)	MAP (mmHg)	HR (bpm)	SPO_2_ (%)	T (°C)
T0	110.8 (12.4)	69.0 (15.7)	97.2 (1.5)	36.3 (0.3)	106.7 (12.8)	70.9 (9.8)	97.4 (1.6)	36.2 (0.5)
T1	97.4 (14.1)	63.0 (11.4)	99.4 (0.9)	36.3 (0.3)	94.8 (16.5)	66.9 (10.3)	99.3 (1.0)	36.2 (0.5)
T2	88.6 (15.7)	56.9 (9.4)	99.6 (0.5)	36.3 (0.3)	86.2 (12.4)	58.5 (9.1)	99.8 (0.6)	36.3 (0.5)
T3	85.8 (11.5)	55.2 (8.8)	99.7 (0.5)	36.5 (0.2)	82.0 (11.2)	54.3 (9.8)	99.6 (1.1)	36.4 (0.5)
T4	81.0 (17.7)	54.7 (10.1)	99.8 (0.4)	36.4 (0.4)	78.6 (10.0)	54.3 (7.5)	99.6 (1.0)	36.4 (0.5)
T5	77.9 (9.7)	53.0 (8.8)	99.8 (0.4)	36.2 (0.4)	76.4 (10.4)	53.1 (6.7)	99.6 (0.8)	36.1 (0.5)
T6	93.9 (12.8)	64.8 (11.4)	99.7 (1.1)	36.9 (0.3)	93.5 (16.5)	65.0 (9.9)	99.7 (0.9)	36.0 (0.4)
T7	104.3 (14.5)	66.4 (9.8)	98.8 (1.6)	36.1 (0.3)	104.3 (11.6)	67.8 (9.7)	98.9 (1.3)	36.0 (0.4)
T8	98.4 (12.0)	67.3 (14.8)	97.9 (1.7)	36.2 (0.4)	98.6 (12.3)	65.5 (11.9)	98.9 (1.8)	36.1 (0.4)
T9	99.4 (12.3)	67.7 (9.5)	97.9 (1.6)	36.3 (0.3)	97.9 (13.1)	65.3 (8.7)	97.5 (2.0)	36.3 (0.3)

Data is presented as mean standard deviations. TEAS: transcutaneous electrical acupoint stimulation. T0: before anesthesia; T1: induction; T2: intubation; T3: onset of surgery; T4: 30 minutes after surgery; T5: at the end of surgery; T6: extubation; T7: out of operation room; T8: at the time of entry to PACU; T9: at the time out of PACU. MAP: mean arterial pressure; HR: heart rate; SPO_2_: oxygen saturation; T: core temperature.

**Table 4:** Evaluation the status of MMSE, PONV, pain, extra analgesia, and QoR-40 scores in patients between two groups.

	TEAS group (*n* = 35)				Control group (*n* = 35)			
	T0	T9	T11	T12	T0	T9	T11	T12
MMSE (scores)	28.1 (1.4)^#^	26.2 (2.8)	28.1 (1.6)^#^	28.3 (1.4)^#^	28.4 (1.7)^#^	25.8 (3.6)	28.1 (1.6)^#^	28.6 (1.3)^#^

	T5	T9	T11	T12	T5	T9	T11	T12
PONV	0	1 (2.9%)	2 (5.7%)	0	0	2 (5.7%)	2 (5.7%)	0
Pain	2 (5.7%)	7 (20%)	8 (22.9%)	3 (8.6%)	1 (2.9%)	6 (17.1%)	8 (19.6%)	7 (20%)
Extra analgesia	3.8 (1.9)^*∗*^				5.0 (2.9)			

	T0	T11	T12		T0	T11	T12
QoR-40 (scores)	199.0 (1.5)	191.7 (4.4)^*∗*^	195.3 (1.9)^*∗*^		198.7 (1.8)	189.1 (4.3)	193.3 (3.0)

Data is presented as mean standard deviation and numbers (proportion). TEAS: transcutaneous electrical acupoint stimulation; MMSE: minimum mental state examination; PONV: postoperative nausea and vomiting. QoR-40: 40-item questionnaire. T0: before anesthesia; T5: at the end of surgery; T9: at the time out of PACU; T11: postoperative 24 hours; T12: postoperative 48 hours. ^#^*P* < 0.001 vs. T9; ^*∗*^*P* < 0.05 vs. control group.

**Table 5:** Serum concentration of biochemical indicators.

	TEAS group (*n* = 35)					Control group (*n* = 35)				
	T0	T4	T5	T9	T10	T0	T4	T5	T9	T10
1L-1*β* (ng/ml)	0.1 (0.6)	0.1 (0.1)	0.1 (0.0)	0.1 (0.0)	0.1 (0.0)	0.1 (0.1)	0.1 (0.1)	0.1 (0.1)	0.1 (0.0)	0.1 (0.0)
TNF-*α* (pg/ml)	0.2 (0.2)	0.2 (0.2)	0.2 (0.2)	0.2 (0.2)	0.2 (0.2)	0.2 (0.2)	0.2 (0.2)	0.2 (0.1)	0.2 (0.1)	0.2 (0.1)
Epinephrine (ng/ml)	0.2 (0.2)	0.2 (0.2)	0.2 (0.2)	0.2 (0.1)	0.2 (0.1)	0.2 (0.3)	0.2 (0.3)	0.2 (0.3)	0.2 (0.3)	0.2 (0.3)
Blood glucose (mmol/L)	5.3 (0.6)	5.2 (0.5)	5.5 (1.0)^#^	5.6 (1.2)^#^	5.7 (1.0)^#^	5.3 (0.5)	5.3 (0.5)	6.2 (2.4)^#^	6.3 (2.4)^#^	5.9 (1.9)^#^
Blood lactic acid (mmol/L)	0.9 (0.4)	1.0 (0.4)	1.0 (0.4)	1.0 (0.4)	1.0 (0.7)	1.0 (0.3)	1.0 (0.3)	1.1 (0.3)	1.1 (0.4)	1.1 (0.6)

TEAS: transcutaneous electrical acupoint stimulation.T0: before anesthesia; T4: 30 minutes after surgery; T5: at the end of surgery; T9: at the time out of PACU. IL-1*β*: interleukin-1; TNF-*α*: tumor necrosis-*α*. ^#^*P* < 0.05 vs. T4.

## Data Availability

Because the data belong to the corresponding author team, some other data have not been publicated. Anyone who needs the data should get the permission from the corresponding author.
